# The Effects of the BAILAMOS Dance Program on Brain Functional Connectivity of Older Latinos: An Exploratory Study

**DOI:** 10.1093/geroni/igaa057.1629

**Published:** 2020-12-16

**Authors:** Guilherme Moraes Balbim, Olusola Ajilore, Melissa Lamar, Kirk Erickson, Susan Aguiñaga, Eduardo Bustamante, David Marquez

**Affiliations:** 1 University of Illinois at Chicago, Chicago, Illinois, United States; 2 Rush University Medical Center, Chicago, Illinois, United States; 3 University of Pittsburgh, Pittsburgh, Pennsylvania, United States; 4 University of Illinois at Urbana-Champaign, Urbana, Illinois, United States

## Abstract

Compared to non-Latinos whites, older Latinos are at higher risk of cognitive impairment and engage in less leisure-time physical activity (PA). Resting-state brain functional connectivity (FC) is a putative biomarker for age-related cognitive decline. PA plays a role in FC of brain networks associated with cognitive decline. Objective: Investigate the effects of the BAILAMOS^™^ dance program on FC in three brain networks associated with age-related cognitive decline (Default Mode [DMN], Frontoparietal [FPN], and Salience [SAL] networks). Methods: Single-group pre-post design. Ten cognitively intact older Latinos participated in the four-month (2x/week for 60min) BAILAMOS^™^ dance program with four Latin dance styles. MRI was obtained pre- and post-intervention. FC was analyzed using the resting-state fMRI toolbox (CONN) via pairwise BOLD signal correlations and then converted into z-scores. We performed dependent t-tests, computed Cohen’s d and 95%CI for p < 0.05. Results: Within-FPN FC significantly increased (t(9) = 2.35, p = 0.043, d = 0.70) from pre (M=0.49±0.15) to post-intervention (M=0.59±0.13). In the DMN, we observed moderate effect size changes in the ratio of the FC between-networks by the FC within-networks (Mdiff = 0.10; 95%CI = -0.01; 0.21, p = 0.08, d = 0.64). Conclusions: The BAILAMOS^™^ program increased within-FPN FC, which is a cognitive-control network related to adaptive control and flexibility. Moderate changes between- vs. within-DMN FC suggest BAILAMOS™ also increased whole-brain DMN integration. Taken together, results might signal that Latin dance can combat the disruption of FC between the DMN and other networks, and within-FPN, which are associated with cognitive decline.

